# Research on Standard Compliance Test Algorithm Based on Electronic Medical Records of Traditional Chinese Medicine Outpatients

**DOI:** 10.1155/2020/8865264

**Published:** 2020-11-03

**Authors:** Li He, Zi Yi Zhou, Fu Sheng Niu, Yu Fan Yang, Qiang Xu, Chuan Biao Wen, Tao Sun, Yue Luo

**Affiliations:** ^1^School of Medical Information Engineering, Chengdu University of Traditional Chinese Medicine, Chengdu, Sichuan, China; ^2^Chengdu University of Traditional Chinese Medicine, Institute of Digital Medicine, Chengdu, Sichuan, China

## Abstract

**Objectives:**

To effectively evaluate the compliance degree between the electronic medical records of Traditional Chinese Medicine (TCM) hospitals, as well as the information platform, and the related information standards of electronic medical records, a standard compliance testing scheme based on electronic medical records of TCM outpatients is proposed.

**Methods:**

This research selected the data of clinical outpatients accumulated in 10 years by the Digital Medicine Institute of Chengdu University of TCM and processed the data through security check and desensitization process. And then 28348 cases of processed electronic medical records of TCM outpatients were inputted into the standard compliance testing platform for assessment. The result was then outputted.

**Results:**

There are 924 cases among the 28348 that can be rated as five-star medical records, 84 cases four-star, 132 cases three-star, 12460 cases two-star, 13488 one-star, and 1260 cases zero-star through the integrity and standardization test.

**Conclusion:**

By the way of assessing the integrity and standardization of data, the standard compliance test algorithm scheme for electronic medical records of TCM outpatients introduced in this paper can solve the problems such as data unavailability caused by ununified codes and incomplete data in the data-sharing process and provides technical support for the construction of data standardization testing in electronic medical records of TCM outpatients.

## 1. Introduction

The Opinions of the CPC Central Committee and the State Council on Deepening the Reform of the Medical and Health System (hereinafter referred to as the Opinions) proposed to establish a practical and shared medical and health information system [1, 2] and employ artificial intelligence technology in promoting the construction of hospital informatization. In order to implement the Opinions, as well as to improve the quality of health services and management, it is urgently needed to establish and improve the health information standard system in our country, to unify information and code standards of various terms in the health field, and to enhance the corresponding exchange and technology standards. In addition, it is of crucial importance to cowork with related departments to strengthen the construction of information standardization and public information service platform [3].

With the deepening of medical and health system reform, the application of electronic medical record has become an inevitable trend of informatization construction in hospitals. In the new medical reform policy, the Ministry of Health and domestic medical institutions have shown certain understanding and attention in the standardization development of electronic medical records and revised successively about 150 health information standards such as the basic dataset of electronic medical record, specification for sharing document of electronic medical record, technical specification for hospital information platform based on electronic medical record [4], and so on. On the one hand, many hospitals have set up information system within hospitals with the development of national Jin Wei project, which laid a solid foundation for the study and application of electronic medical record in China; on the other hand, with the development of hospital HIS system in the direction of clinical information system (CIS), electronic medical record is getting more and more attention and a professional committee on it has been set up in China. At the same time, studies on standard compliance test methodology have appeared successively in scientific research filed. In light of the rapid development of computer network technology, it is of great value to transfer electronic medical records with the help of Internet technology. While at present, the development of electronic medical record in China has just started and the research related to compliance test for the electronic medical record digitization of TCM is yet to come. TCM is an important component of medical and healthcare, it is of great urgency to study the compliance test for electronic medical records of TCM digitization.

Therefore, this paper introduces the algorithm of standard compliance test into the health informatization construction of electronic medical records of TCM outpatients and tries to explore the specific medical and health informatization construction that fits China's national conditions. It has great guiding significance to the successful implementation of the standard compliance test for TCM electronic medical record. It can boost the application of information standards of TCM electronic medical records in our country, as well as promote the construction of efficient, unified, data-shared health informatization.

With the popularization of TCM electronic medical record system, the application of standards of TCM electronic medical records has become an important issue to be studied. And the research on standard compliance test for TCM electronic medical records information makes up the lack of health informatization construction and evaluates the application of information standards.

In addition to the characteristics of general electronic medical record, the electronic medical record of TCM outpatient has its own particularity, such as its unique contents, structure, and clinical information standardization [5]. TCM is different from Western medicine in dealing with clinical data from the aspects of disease classification, treatment, test, and so on. For example, TCM pays attention to holistic, dynamic, and personalized diagnosis and syndrome differentiation treatment, and clinical treatment combines four diagnoses, focusing more on the diagnosis and treatment of patients' overall function of their viscera, while Western medicine has a variety of biochemical and physical tests along with clear indicators of diagnostic results. Therefore, the electronic medical records of Western medicine are structured electronic medical records, while the electronic medical records of TCM are semistructured or unstructured. In light of these differences, the assessment for TCM electronic medical records' data as the test object can not only improve their integrity and standardization, but also the research on standard compliance test for TCM electronic medical records can test the application of the standard compliance testing platform, as well as verify whether the application of TCM electronic medical records' information standards is effective, normalized, and standardized.

## 2. Related Researches

**Figure 1:**
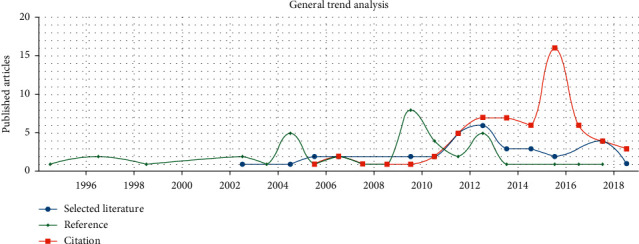
Overall research on standard compliance testing platform.

This study retrieved related research from the database of China National Knowledge Infrastructure (CNKI) with “standard compliance testing platform” as the searching key words and without requirements for the publishing time. There are only 31 articles that are found related to “standard compliance testing platform,” and these articles include “A Framework of SQL Conformance Test” by Li et al. [6] in 2003, “XML-Based Standards Compliance Testing Scheme” by Wu and Fan [7] in 2012, “Research and Development of Health Information Standard Conformance Test Case Library” by Xiao Youhua et al. [8] in 2015, and “Research on Conformance Testing of Data Standard” by Zhu et al. [9] in 2019. Among these studies, there are only 2 that are related to TCM electronic medical records, which is extremely rare. These two articles were published by Si Tong and Wang Wenjing, a master student in Hubei University of Chinese Medicine in 2014 as her graduation dissertation [10, 11]. Si Tong reviewed the current development of TCM electronic medical records in both home and abroad and then proposed and designed an outpatient electronic medical record software with Chinese traditional medicine characteristics [12], which presented a novel method for the development of TCM electronic medical records. While Wang's research was mainly related to the study of standard compliance of TCM electronic medical record information. Three articles are related to electronic health records, which mainly involve the study, proposition, and design of standard compliance test for electronic health records [13–15]. The searching result also shows that there are about 22 articles on standard compliance test, with contents covering from data element standards, platforms, e-commerce products, and e-government data exchange to ODBC standard testing framework [8, 16–28]. It indicates that the research on standard compliance testing platforms is in-depth in testing various types of standards, but short in the medical industry, such as TCM electronic medical records and electronic health records. In addition, from the overall trend of the research as shown in Figure 1, it can be seen that the development of research on the standard compliance testing platform is slow, or even stagnant.

In the era of rapid development of medical informatization, TCM hospitals have accumulated a large number of electronic medical records data. But the existing standard compliance testing and research platforms have their own specific standards, respectively, which leads to the fact that the tested data are not mutually relevant. Therefore, how to effectively utilize these data to improve medical and health service is of vital importance at present [29].

As shown in Figure 1, the selected literatures in this paper were mainly published in the year from 2003 to 2019 in CNKI, with an increase in the number of articles published since 2003. The number of researches on standard compliance testing platform reached a peak between 2012 and 2013. The above figure also shows that the number of citations began to rise in 2005, reaching its peak in 2016 followed by a decline. The references of this paper were published as early as the 1990s or even earlier, which signifies that the topic of this paper appeared as early as back then, providing basic support and basis for current research, and that the research on standard compliance testing platforms has always been a hot issue which needs to be excavated deeply and widely.

This study starts from the literature review, which indicates that the research on the standard compliance test for TCM electronic medical records is rare and lacks available publicly labeled datasets [29]. In view of the fact that the research on entity standardization of electronic medical records being a hotspot driven by international public evaluation tasks^1^, this paper then comes up with a research scheme for standard compliance test based on TCM electronic medical records. The electronic medical records data employed in this paper were randomly selected from the clinical outpatient data accumulated by the Institute of Digital Medicine of Chengdu University of TCM in the past 10 years. The basic four diagnosis and prescription information of TCM outpatients were first collected and processed through data security check and desensitization and then were inputted into the standard compliance testing platform, and the platform outputted the compliance test results subsequently. This research is believed to effectively promote the standardization of TCM outpatient electronic medical records and further bring positive impact on the medical cause.

## 3. Algorithm of Standard Compliance Test for Electronic Medical Records of TCM Outpatients

The two most representative evaluation tasks are ShARe/CLEF eHealth Shared Task 1b [30] in 2013 and SemEval Task 7 [31] in 2014, whose tasks are to find out the coding of entities in electronic medical records such as diseases and symptoms in Systematized Nomenclature of Medicine-Clinical Terms, abbreviated as SNOMED-CT [32].

The algorithm of standard compliance test based on electronic medical records of TCM outpatients includes score assessment algorithm and star-rating assessment algorithm.

### 3.1. The Score Assessment Algorithm of the Standard Compliance Test

The score assessment algorithm is employed to check the integrity and coding standards of the electronic medical record data of TCM outpatients uploaded by medical institutions every single time and then evaluate its quality through certain rules. The score assessment algorithm consists of three parts, namely, integrity assessment, coding standard assessment, and total score assessment. Integrity test and coding standard test are targeted at the assessment of the integrity and coding standards of TCM outpatients' electronic medical records, while the total score is the sum of integrity assessment score and coding standard assessment score calculated according to their weight proportion.

The integrity assessment checks whether the mandatory items in TCM outpatients' electronic medical records are filled in. For example, the name and age of the patient must be put down, and if not, this standard will be scored zero. The mandatory items in TCM outpatients' electronic medical records determined in this study are shown in Table 1.

Coding standard assessment tests whether the codes of TCM symptoms, syndrome types, and treatments in TCM electronic medical records conform with related coding standards. The codes and their corresponding coding standards of TCM outpatients' electronic medical records determined in this study are shown in Table 2.

The integrity assessment algorithm, coding standard assessment algorithm, and total score assessment algorithm are, respectively, introduced as follows:

1. Integrity assessment algorithm
2. Provided that *X*_*i*_ is the number of missing items required in each medical record, *n* is the total number of medical records, *k* is the number of integrity items, and the integrity assessment score is *S*_*i*_, the calculation of *S*_*i*_ is presented as

(1) $$\begin{array}{c} S_{i}=\left(1-\sum_{1}^{n}\frac{X_{i}}{n*k}\right)*100. \end{array}$$

(2) Coding standard assessment algorithm
  Provided that *Y*_*j*_ is the number of missing items, *n* is the total number of medical records, *k* is the number of coding items, and the coding standard assessment score is *S*_*c*_, the calculation of *S*_*c*_ is shown as

(2) $$\begin{array}{c} S_{c}=\left(1-\sum_{1}^{n}\frac{Y_{j}}{n*k}\right)*100. \end{array}$$

(3) Total score assessment algorithm
  The total score of TCM electronic medical record is codetermined by the integrity assessment score and the coding standard assessment score. Provided that the total score is *S*_*t*_, the calculation of *S*_*t*_ is stated as

(3) $$\begin{array}{c} S_{t}=S_{i}*0.5+S_{c}*0.5. \end{array}$$

### 3.2. The Star-Rating Assessment Algorithm

#### 3.2.1. Star-Rating Assessment of Each Outpatient Electronic Medical Record

It is a process to star-rate every medical record uploaded by medical institutions via star-rating assessment algorithm. The star-rating process is conducted as per the standards described in Table 3, after the assessment of the integrity and coding standard of contents in medical records.

The following star-rating principles are indicated from Table 3:

1. Medical records will be rated as one-star records if the above standards from no.1 to no.5 are met simultaneously.
2. Medical records will be rated as two-star records if the above standards from no.1 to no.7 are met simultaneously.
3. Medical records will be rated as three-star records if the above standards from no.1 to no.9 are met simultaneously.
4. Medical records will be rated as four-star records if the above standards from no.1 to no.11 are met simultaneously.
5. Medical records will be rated as five-star records if the above standards from no.1 to no.14 are met simultaneously.

#### 3.2.2. Overall Star-Rating Assessment of Outpatient Medical Records Uploaded by Medical Institutions in a Single Time

The overall star-rating assessment of medical records uploaded by medical institutions every single time is composed of the star-ratings of the five-star, four-star, three-star, two-star, and one-star medical records as shown in Formula (4).

## 4. The Application of Standard Compliance Testing Platform for TCM Electronic Medical Records

Based on the above algorithm, the setting up of standard compliance testing platform for TCM electronic medical records is achieved via the applying of Visual Studio 2012 development tools, C# programming language, and SQL Server 2008 database management system.

**Figure 2:**
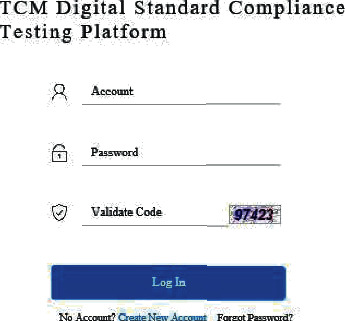
Login interface of digital standard compliance testing platform for TCM.

**Figure 3:**
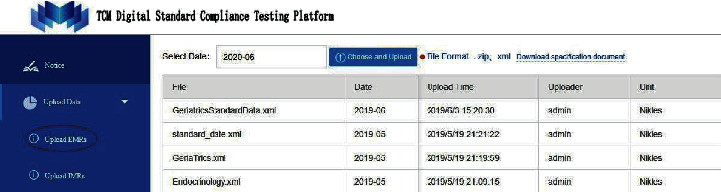
Interface of uploading outpatient medical record data by medical institutions.

After registering and logging in the standard compliance testing platform for TCM electronic medical records, as shown in Figure 2, medical institutions can upload outpatient electronic medical records by clicking the “Upload outpatient medical record” button under the menu of “Upload data,” as shown in Figure 3.

The data of clinical outpatients accumulated in 10 years by the Digital Medicine Institute of Chengdu University of TCM were selected to be dealt with through security check and desensitization process. After processing, 28348 cases of electronic medical records of TCM outpatients were inputted into the standard compliance testing platform for assessment. The result was then outputted.

## 5. Result

**Figure 4:**
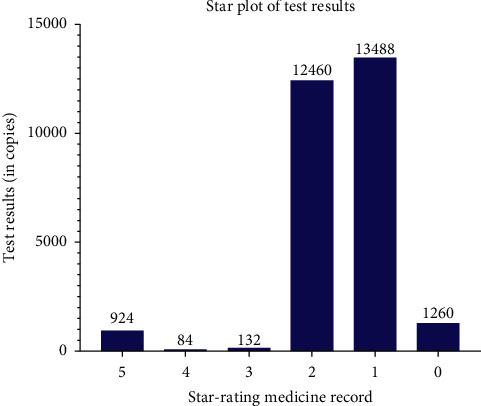
Star-rating of tested data.

The result of testing shows that in all the 28348 cases of TCM outpatient electronic medical records, there are 924 cases are rated as five-star, 84 cases four-star, 132 cases three-star, 12460 cases two-star, and 1260 cases zero-star, as shown in Table 4 and Figure 4.

**Figure 5:**
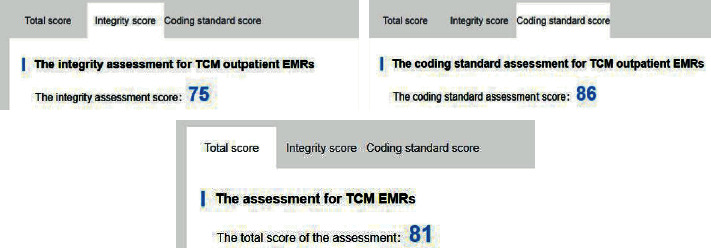
Scoring results of TCM outpatient electronic medical record case.

As a result of the integrity assessment and coding standards assessment, the integrity score and coding standard score of the TCM outpatient electronic medical records case data upload this time are 75 points and 86 points, respectively, and the total score is 81 points, as shown in Figure 5.

100 cases of TCM outpatient medical records were randomly selected manually and assessed by the above scoring and star-rating algorithm designed in this paper, and the result is consistent with the above result calculated by the standard compliance testing platform.

## 6. Conclusion

The algorithm of standard test for TCM outpatient electronic medical records put forward in this paper assesses outpatient electronic medical records from the aspects of integrity and coding standard while combining computer technology and traditional Chinese medicine. It presents a monitoring platform for the standard writing of TCM outpatient electric medical records, as well as a solution to problems such as data unavailability caused by disunified codes and incomplete data in the sharing process. Additionally, it gives feedbacks on the quality of data to hospitals which provide their electronic medical record data in order to correct the data timely and provides technical support for the construction of data standardization testing of TCM outpatient electronic medical records.

## Figures and Tables

**Table 1:** Mandatory items in TCM outpatient electronic medical records: number, item, and standard.

Number	Item	Standard
1	Location	Cannot be blank
2	Pathological nature	Cannot be blank
3	Four diagnosis	At least one
4	TCM symptom code	At least one
5	TCM symptom	At least one
6	TCM diagnosis	At least one
7	TCM syndrome type	At least one
8	TCM syndrome type code	At least one
9	TCM treatment	At least one
10	TCM treatment code	At least one
11	Name	Cannot be blank
12	ID number	Cannot be blank
13	Age	Cannot be blank
14	Gender code	Cannot be blank
15	Identification of dominant diseases in TCM	Cannot be blank
16	Code of dominant diseases in TCM	At least one

**Table 2:** Codes and standards of TCM outpatient electronic medical records.

Number	Item	Standard	Source of coding standard
	Ethnicity code	Within code range	People's Republic of China health industry standards WS 445.11-2014
	Code of medical insurance type	Within code range	People's Republic of China health industry standards WS 445.11-2014
	Level of disease severity	Within code range	People's Republic of China health industry standards WS 445.11-2014
	Biological gender code	Within code range	People's Republic of China health industry standards WS 445.11-2014
	Code of ID type	Within code range	People's Republic of China health industry Standards WS 445.11-2014
	Code of dominant diseases in TCM	Within code range	People's Republic of China health industry standards WS 445.11-2014
	Type of diagnosis	Within code range	People's Republic of China health industry standards WS 445.11-2014
	Code of payment method	Within code range	People's Republic of China health industry standards WS 445.11-2014
	Nationality code	Within code range	People's Republic of China health industry standards WS 445.11–2014

1	Clinic terminology of traditional Chinese medical diagnosis and treatment-Therapeutic methods	Within code range	National standard of the People's Republic of China [33]

2	Clinic terminology of traditional Chinese medical diagnosis and treatment-Syndromes	Within code range	National standard of the People's Republic of China [34]

3	Clinic terminology of traditional Chinese medical diagnosis and treatment-Diseases	Within code range	National standard of the People's Republic of China [35]

4	Classification and codes of diseases and Zheng of traditional Chinese medicine	Within code range	National standard of the People's Republic of China [36]

**Table 3:** Star-rating standards for each medical record.

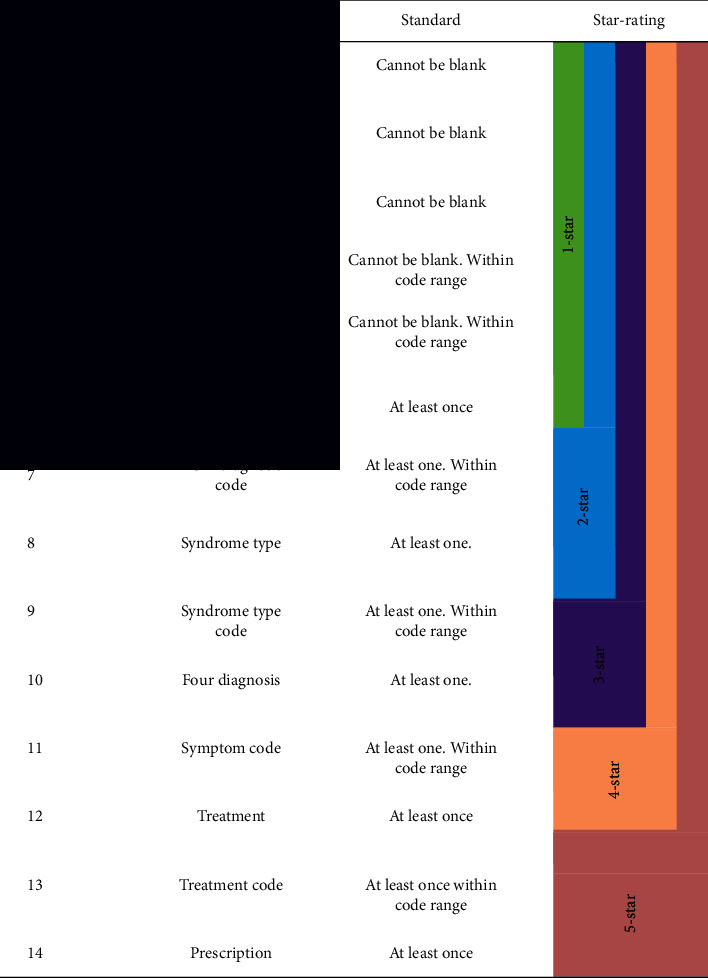

**Table 4:** Test result of the application case.

Name of institution tested	Data content tested	Test result
Digital Medicine Institute of Chengdu University of TCM	Basic information of patients four diagnosis prescription	5-Star medical records: 924
		4-Star medical records: 84
		3-Star medical records: 132
		2-Star medical records:12460 1-star medical records: 13488
		0-Star medical records: 1260

Total		28348

## Data Availability

The basic data of our study come from the Institute of Digital Medicine of Chengdu University of Traditional Chinese Medicine. Through the cleaning, desensitization, and privacy security processing of the basic data, a total of 28348 data are obtained. And the basic data content can be obtained through the relevant platform of the Digital Medicine Research Institute of Chengdu University of Traditional Chinese Medicine.
